# Incorporating Conservation Zone Effectiveness for Protecting Biodiversity in Marine Planning

**DOI:** 10.1371/journal.pone.0078986

**Published:** 2013-11-04

**Authors:** Azusa Makino, Carissa J. Klein, Maria Beger, Stacy D. Jupiter, Hugh P. Possingham

**Affiliations:** 1 Australian Research Council Centre of Excellence for Environmental Decisions, School of Biological Sciences, The University of Queensland, Brisbane, Queensland, Australia; 2 Fiji Country Program, Wildlife Conservation Society, Suva, Fiji; 3 Department of Life Sciences, Imperial College London, Ascot, Berkshire, United Kingdom; Leibniz Center for Tropical Marine Ecology, Germany

## Abstract

Establishing different types of conservation zones is becoming commonplace. However, spatial prioritization methods that can accommodate multiple zones are poorly understood in theory and application. It is typically assumed that management regulations across zones have differential levels of effectiveness (“zone effectiveness”) for biodiversity protection, but the influence of zone effectiveness on achieving conservation targets has not yet been explored. Here, we consider the zone effectiveness of three zones: permanent closure, partial protection, and open, for planning for the protection of five different marine habitats in the Vatu-i-Ra Seascape, Fiji. We explore the impact of differential zone effectiveness on the location and costs of conservation priorities. We assume that permanent closure zones are fully effective at protecting all habitats, open zones do not contribute towards the conservation targets and partial protection zones lie between these two extremes. We use four different estimates for zone effectiveness and three different estimates for zone cost of the partial protection zone. To enhance the practical utility of the approach, we also explore how much of each traditional fishing ground can remain open for fishing while still achieving conservation targets. Our results show that all of the high priority areas for permanent closure zones would not be a high priority when the zone effectiveness of the partial protection zone is equal to that of permanent closure zones. When differential zone effectiveness and costs are considered, the resulting marine protected area network consequently increases in size, with more area allocated to permanent closure zones to meet conservation targets. By distributing the loss of fishing opportunity equitably among local communities, we find that 84–88% of each traditional fishing ground can be left open while still meeting conservation targets. Finally, we summarize the steps for developing marine zoning that accounts for zone effectiveness.

## Introduction

Marine spatial planning that incorporates multiple conservation zones in a marine protected area (MPA) network provides planners and policy-makers with more flexibility to accommodate the objectives of multiple users compared with a traditional two-zone planning process (*i.e.* reserve zones versus non-reserve zones) [1]. Different types of zones within a management scheme afford varying degrees of protection for biodiversity depending on the degree of restriction of human use. For example, in the Great Barrier Reef Marine Park in Australia, the “General Use Zone” is the least restricted zone and allows activities such as mining, tourism, fishing, mariculture, and shipping, whereas the “Preservation Zone” is completely no access [2]. Establishing multiple zones in a MPA network may improve the overall effectiveness of the MPA network by minimizing costs to, and conflict between, different activities (*e.g.* conservation versus fishing) [3], [4]. Furthermore, well- designed and managed MPA networks will benefit both biodiversity and fisheries by providing many direct (*e.g.* restoring fish populations) and indirect benefits (*e.g.* abating threats from overfishing that can cause coral degradation, spill over benefit for fishery) [5]–[8].

Marine zoning approaches have been developed and used around the world. In the Great Barrier Reef Marine Park in Australia, managers decided on the location of different zones on the basis of extensive stakeholder consultation, and the spatial allocation of no-take reserves were informed using Marxan, a systematic conservation planning tool [2], [9]. A more advanced version of this tool, Marxan with Zones, has been used to inform the allocation of zones for fishery and biodiversity objectives in California, U.S.A [10], the Caribbean [11], and Raja Ampat, Indonesia [12]. Other methods for systematic marine zoning are to use multi-criteria decision analysis frameworks to incorporate opinions of different stakeholders (*e.g.* Villa *et al*. [13] and Portman [14]), develop a GIS- based model (*e.g.* Bruce and Eliot [15]), or using a web-based zoning platform [16]. Although ecological effects were mentioned in the use of multi-criteria method by Lahdelma et al. [17], none of these studies on marine zoning incorporate how different kinds of zones differentially contribute to the conservation of various aspects of biodiversity (henceforward “zone effectiveness”). We focus only on the ecological effectiveness defined as the relative contribution of actions to realizing conservation objectives and not the management effectiveness that is based on both human behavior and species' ecology [18]. While most managers implicitly realize that less restrictive zones will be less effective for biodiversity conservation which is also quantified [19], planners typically assume that all zones are either completely effective or completely ineffective when calculating performance against biodiversity targets.

Incorporating zone effectiveness into the design of marine zoning for a MPA network requires quantitative data on the effectiveness of each zone at protecting each species or habitats. Obtaining such information is challenging as we know very little about zone effectiveness *in situ* because data are costly and time-consuming to acquire [20]. In general, permanent closure zones are recognized as ecologically effective because they prohibit extractive activities, which provide well known benefits to biodiversity [21]. Other types of zones contribute differently to the protection of biodiversity, depending on the kinds of activities allowed and their intensity [22], [23]. For example, high fishing pressure threatens marine biodiversity [24]. Different zones regulating fishing activities include temporal closures, gear prohibitions, species-specific bans, and quotas [2]. These actions for conservation are not equally effective at protecting biodiversity [25]. Furthermore taxonomic groups with different types of life histories or ecological characteristics will be affected differently by different kinds of zones [21]. However, ecological effects of different types of conservation zone on different taxa are poorly understood and quantified relative to permanent closure zones.

Quantifying the relative costs of each zone is also a critical component to zoning as it helps planners produce plans that minimise negative socioeconomic impacts (*e.g.* regulating fishing activities in a way that would reduce the opportunity cost of foregone fishing). In addition to zone effectiveness, the relative cost of different conservation zones can impact the location and size of each zone [10]. Although there are many possible conservation costs (*e.g.* acquisition costs, management costs, transection costs, etc. Neidoo et al. [26]) most marine planners only consider opportunity costs [27], which can differ between zones in a given place, depending on which fishing activities are prohibited in the zone [10], [12]. For example, the opportunity cost of a permanent closure zone that prohibits all fishing activities will be higher than in a partial protection zone that allows some kinds of fishing. Data on the opportunity cost of different zones are rarely available for marine planning (but see Klein *et al.* [10]), and can be costly to collect, especially over large planning regions [28]. To compensate, planners often develop surrogates to represent the opportunity cost of implementing a no-take area based on population [29], area [30], threats [31], and etc. However, it is unclear how these cost surrogates can be applied to other types of conservation zones. Given this uncertainty, we explore how the relative cost difference of permanent closure zones and partial protection zones impacts conservation plans. The consideration of opportunity costs is just one way to address fishing interests in marine planning. Another way is to ensure that a designated or even proportion of fishing grounds for different fisheries [10] or communities [12] are represented in a fishing zone [32].

We test our methodology in a spatial prioritization that considers two different conservation zones (permanent closure and partial protection) and one fishing zone (open zone) in Fiji under five different planning scenarios, where we varied the effectiveness of the partial protection zone, relative zone costs, and targets for fishing communities. We specifically selected Fiji as a case study because zone effectiveness score were previously developed by local experts [18]. Zone effectiveness was designed specifically for looking at the effectiveness of Fiji's MPA network at achieving the Fiji government biodiversity target to effectively protect 30% of marine areas by 2020, as well as the Aichi biodiversity target 11. We evaluate the results from the scenarios to determine: (1) the effects on spatial priorities using different levels of zone effectiveness and different zone costs; and (2) the maximum proportions of each fishing ground that can be left open while achieving conservation targets for a minimum cost.

## Methods

### Study region

**Figure 1:**
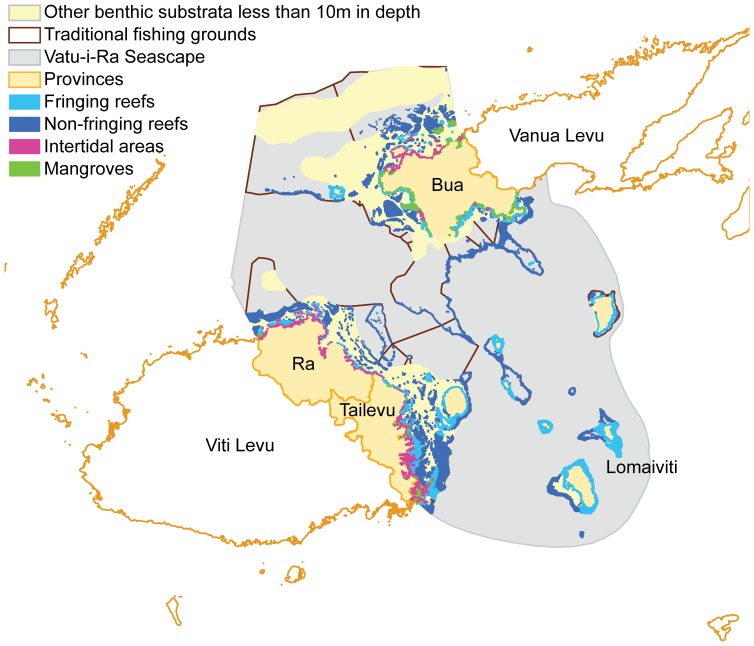
Study region and habitat maps of conservation features. Our study region is the traditional fishing grounds of four provinces (*i.e.* Ra, Tailevu, Lomaiviti and Bua) along the coastline of the Vatu-i-Ra Seascape, Fiji. Conservation features were (1) fringing reefs, (2) non-fringing reefs, (3) mangroves, (4) intertidal areas, and (5) other benthic substrata less than 10 m in depth.

We focused our study on the traditional fishing grounds in the Vatu-i-Ra Seascape in Fiji, which includes the inshore marine and coastal habitats of the four provinces of Ra, Tailevu, Lomaiviti and Bua (Fig. 1). Fiji's traditional fishing grounds (*i qoliqoli*) are legally demarcated by the iTaukei Land and Fisheries Commission (the traditional fishing grounds areas of the four provinces are shown in Fig. 1). The traditional fishing grounds extend to the edge of the furthest reef crest (fringing, barrier or patch) away from districts that have traditional fishing rights in the area (Fig. 1). Within these areas, indigenous Fijian maintain customary fishing access rights and have some ability to designate management zones, with support from co-management partners in the Fiji Locally Managed Marine Area network [33]. The Fiji locally managed marine areas are equivalent to MPAs although they aim for the sustainable use of the marine areas and not for conservation *per se*. They are governed by a group of local resource management and conservation practitioners [18]. In this study, we used a broader definition of MPAs that includes locally managed marine areas, as they also contribute to the protection of biodiversity. In Fiji, there are permanent closure zones where all extractive activities are prohibited and periodically harvested zones [18]. Some of the periodically harvested closure zones have a controlled harvest once per year, but many of them do not have a well-developed management plan for harvesting that specifies frequency and duration [18], [34].

### Spatial prioritization

We used the systematic planning tool Marxan with Zones, which is a decision-support tool for selecting priority areas for zoning marine or terrestrial areas to achieve conservation and/or fishing targets for minimum cost. Marxan with Zones minimizes the total cost of a zoning system, which is the sum of the costs of placing each planning unit into a particular zone subject to meeting target amounts of conservation and/or fishing features [35]. Marxan with Zones allows users to set how much each zone will contribute towards meeting conservation and/or fishing targets - zone effectiveness [35]. Specifically, Marxan with Zones solves the mathematical problem:
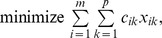


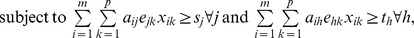
where *m* is the total number of planning units (*i* = 1, …, *m*), *p* is the total number of zones (*k* = 1, …, *p*), and 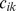 is the cost of allocating planning unit *i* to zone *k*. For all planning units (*i* = 1, …, *m*) and zones *k* (*k* = 1, …, *p*), 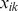 is a member of {0, 1} and if planning unit *i* is allocated as zone *k*, 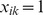 and if not 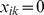. A planning unit *i* cannot be allocated to more than one zone, thus 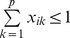. The first constraint is to meet the conservation target while applying zone effectiveness: 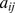 is the amount of conservation feature *j* (*i* = 1, …, *n*) in planning unit *i*, and 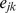 is the zone effectiveness of zone *k* to conservation feature *j*. The target amount for conservation feature *j* is defined as 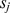. The second constraint is to meet the target amount for the fishing feature *h* (*h* = 1, …, *q*), defined as 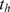. The amount of the fishing feature *h* in planning unit *i* is 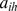 and 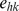 is the zone effectiveness of zone *k* to meet the target of the fishing feature *h*.

### Conservation targets and planning units

We considered five different marine habitats in the region as conservation features: fringing reefs, non-fringing reefs, mangroves, intertidal areas, and other benthic substrata less than 10 m in depth (Fig. 1) [18]. Intertidal areas include mudflats but exclude the mangroves. Areas less than 10 m in depth where there are no fringing reefs, non-fringing reefs, mangroves or intertidal areas are classified as other benthic substrata less than 10 m in depth. In addition to conservation features, we also had fishing features, which are the traditional fishing grounds in the study region (n = 27). For the conservation features, we aimed to protect 30% of the distribution of each feature in either a permanent closure or partial protection zone [18]. The conservation targets were in accordance with national targets and outputs from an expert workshop in Fiji [18]. For fishing features, we aimed to evenly include as much of each traditional fishing ground in the open zone without compromising the conservation targets. We did this by gradually increasing the target for fishing features until a conservation target was not achieved. Not all features were targeted for all planning scenarios, described in the subsection of “5. Planning scenarios” below.

We overlaid 1 km^2^ hexagonal planning units on the study region (n = 10044). We only included traditional fishing grounds in our planning region that were larger than 20 km^2^ and had no more than 10% of their total area in closures from fishing. We excluded small traditional fishing grounds since they do not always contain conservation features (*i.e.* habitats) and/or were too small to allocate multiple zones. Each planning unit could be allocated to one of the three zones to create a MPA network except for existing permanent closure zones and periodically harvested closure zones. Existing permanent closure zones and periodically harvested closure zones in the study region were assigned to the permanent closure zone and the partial protection zone, respectively, for all analyses. Conservation features within those existing conservation areas contributed towards the overall conservation targets in every analysis.

### Zones effectiveness and costs

We considered three zones: (1) a permanent closure zone, which offers the highest level of protection; (2) a partial protection zone, which may be periodically harvested for fisheries resources; and (3) an open zone, under which all activities are permissible and we assume it offers no protection for biodiversity. A partial protection zone has no predetermined harvest frequency, intensity or duration (referred to as “periodic closure with uncontrolled harvesting” zones in Mills *et al*. [18]). Experts determined the effectiveness of zones at protecting selected species groups (*e.g.* corals, targeted invertebrates, non-targeted invertebrates, targeted fish, non-targeted fish, and coralline algae in fringing reef habitat) that they considered of national importance in five marine habitats in our study region, Fiji, working under the assumptions that permanent closure zones offer the highest benefit for biodiversity, while periodic harvests reduce zone effectiveness [18]. We chose to use the more conservative measures of zone effectiveness for the partial protection zones by adopting the measures defined in Mills *et al.* [18] for periodically harvested areas in an uncontrolled manner. The effectiveness of any zone is a real number between 0 and 1 inclusive, where 0 is completely ineffective and 1 is 100% effective in meeting targets in that zone. Thus, the zone effectiveness, 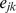, for permanent closure zones at protecting conservation feature *j* was 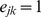 (maximum effectiveness), and for open zones at protecting conservation feature *j* was 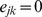
(completely ineffective) for every conservation feature. For partial protection zones (referred to as “periodic closure with uncontrolled harvesting” zones in Mills *et al*. [18]) at protecting conservation feature *j*, the highest, average, and lowest zone effectiveness values determined by local experts in Fiji were used (Table 1) [18]. For fishing feature *h*, the zone effectiveness for the open zone was 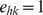, and 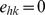 for the permanent closure and partial protection zone.

**Table 1:** Zone effectiveness for the partial protection zone at achieving conservation targets, as determined by experts in Fiji (referred to as “periodic closure with uncontrolled harvesting” zone in Mills *et al*. [18]).

	Scenario			
Conservation Features	(1) Equal	(2) Highest	(3) Average	(4) Lowest
Fringing reefs	1	0.6	0.39	0.1
Non-fringing reefs	1	0.8	0.46	0.1
Mangrove	1	0.85	0.56	0.1
Intertidal	1	0.8	0.48	0.1
Other benthic less than 10 m	1	0.8	0.6	0.3

Zone effectiveness at meeting conservation targets for the permanent closure zone was always 1 (fully effective), and for the open zone was always 0 (completely ineffective) for all scenarios. Scenario 5 used the same average zone effectiveness as scenario 2.

We estimated the opportunity costs of fishing using the fishing pressure data modeled by Klein *et al*. [29] for the permanent closure zone. The approach assumed that fishing is allowed on all coral reefs. This assumption was reasonable as all reefs are open access for subsistence use under the present Fiji Fisheries Act (1942). They estimated fishing pressure to be proportional to the number of people living within 35 km of each planning unit on the basis that fishing pressure is roughly correlated with the coastal population in Fiji [29], [36]. This was used as there is no existing comprehensive fishing information available for Fiji and the global datasets did not adequately represent fishing pressure in the study region [24], [37]. We assigned the relative costs of partial protection zones as 75%, 50% and 25% of the cost of the permanent closure zones since there was no information how the opportunity costs of fishing would scale among conservation zones in Fiji. The opportunity cost for an open zone was zero since there were no restrictions on fishing.

### Planning scenarios

We considered five different planning scenarios, where we varied the effectiveness of the partial protection zone, relative zone costs, and targets for fishing communities. In scenario 1, we considered partial protection zones to be as effective as permanent closure zones (equal zone effectiveness) at meeting conservation targets to represent cases where locally managed partial protection zones are highly effective in protecting biodiversity [38], [39] (Table 2). In scenario 2-4, we applied the highest (scenario 2), average (scenario 3) and lowest (scenario 4) zone effectiveness scores to the partial protection zones (Table 1 and 2) at meeting conservation targets using the consensus values from the expert workshop [18]. In scenario 5, we treated each traditional fishing ground as a fishing feature to include in a zone and targeted them the same as we targeted conservation features. We increased the targeted amount of fishing features until conservation target became impossible to achieve, to find the highest amount of traditional fishing grounds that can remain open while still meeting the conservation targets. In our study region, the traditional fishing grounds of four provinces include existing permanent closure or partial protection zones but we only allowed the fishing feature to be achieved in the open zone. The five scenarios were analyzed for each of the three different zone costs for partial protection zones (75%, 50% and 25% of the cost of the permanent closure zones) as described in subsection 2.4. For each scenario, we ran Marxan with Zones 100 times.

**Table 2:** Description of scenarios using different zone effectiveness values, 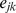, zone effectiveness of zone *k* (*k* = 3) for conservation feature *j* (*j* = 5) and 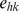 for fishing feature *h* (*h* = 27).

Scenario	Description
1	Partial protection zones are as effective as permanent closure zone for all conservation features to achieve conservation targets. 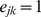 for the permanent closure zone, 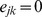 for the open zone, 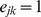 for the partial protection zone.
2	Uses the highest zone effectiveness for partial protection zone. 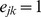 for the permanent closure zone, 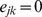 for the open zone, 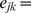 highest for the partial protection zone (see Table1).
3	Uses the average zone effectiveness for the partial protection zone. 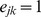 for the permanent closure zone, 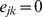 for the open zone, 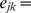 average for the partial protection zone (see Table1).
4	Uses the lowest zone effectiveness for the partial protection zone. 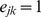 for the permanent closure zone, 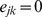 for the open zone, 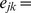 lowest for the partial protection zone (see Table1).
5	Uses the average zone effectiveness and aims an equitable amount of fishing grounds remained open for fishing. 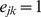 for the permanent closure zone, 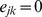 for the open zone, 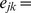 average for the partial protection zone (see Table1). For fishing feature *h*, 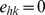 for the permanent closure zone, 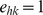 for the open zone, 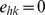 for the partial protection zone

Zone effectiveness was the same for all conservation features as well as for all fishing features. Fishing features were targeted only in scenario 5.

In order to test whether solutions for the 4 scenarios that used different degrees of zone effectiveness and costs differ statistically, we conducted model-based clustering. We combined all solutions of scenarios 1–4 and assigned an unknown structure. We then classified solutions into clusters that are cohesive and separated from other clusters in multivariate space. Each solution contained information of the planning units that were allocated to the permanent closure and partial protection zones. We used multivariate normal mixture modeling that evaluated alternative numbers of clusters as most likely models to determine the statistically appropriate classification of the solutions among scenarios 1 to 4 (*i.e.* 100 solutions per scenario). The function “mclustBIC” in R package mclust was applied to implement the modeling [40], [41]. We tested these models with the Bayesian Information Criterion (BIC). BIC provides an approximation to the Bayes factor, allowing comparison of a model with differing numbers of clusters [41]. BIC is defined as twice the log likelihood in mclust, unlike as for ordinal BIC. Thus, a high value of BIC indicates stronger evidence for a certain number of clusters [41]. We used a sub-model of VEV: volumes of all clusters as varying (V), shapes of all clusters as equal (E), and orientation of all clusters as varying (V) to identify the number of clusters across all solutions between scenarios (n = 400) with the highest BIC.

We compared the best available solution, which was the least costly solution that met the targets out of the 100 runs, from scenario to scenario as well as the “selection frequency,” which was how many times each planning unit was selected out of 100 runs. We used the best ten solutions to get average values for the total costs.

## Results

**Figure 2:**
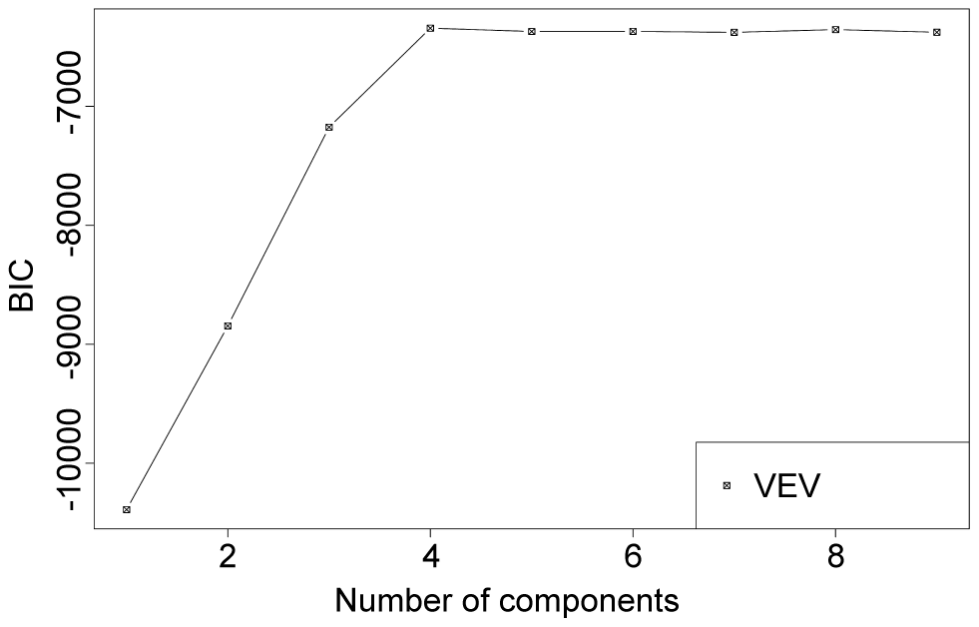
Result of classification of the solutions using multivariate normal mixture modeling. We used the sub-model of VEV (volumes of all clusters as varying (V), shapes of all clusters as equal (E), and orientation of all clusters as varying (V)) to identify the number of clusters across all solutions between scenarios (n = 400) with the highest Bayesian Information Criterion (BIC). BIC is defined as twice the log likelihood in this method [41], unlike as for ordinal BIC.

**Figure 3:**
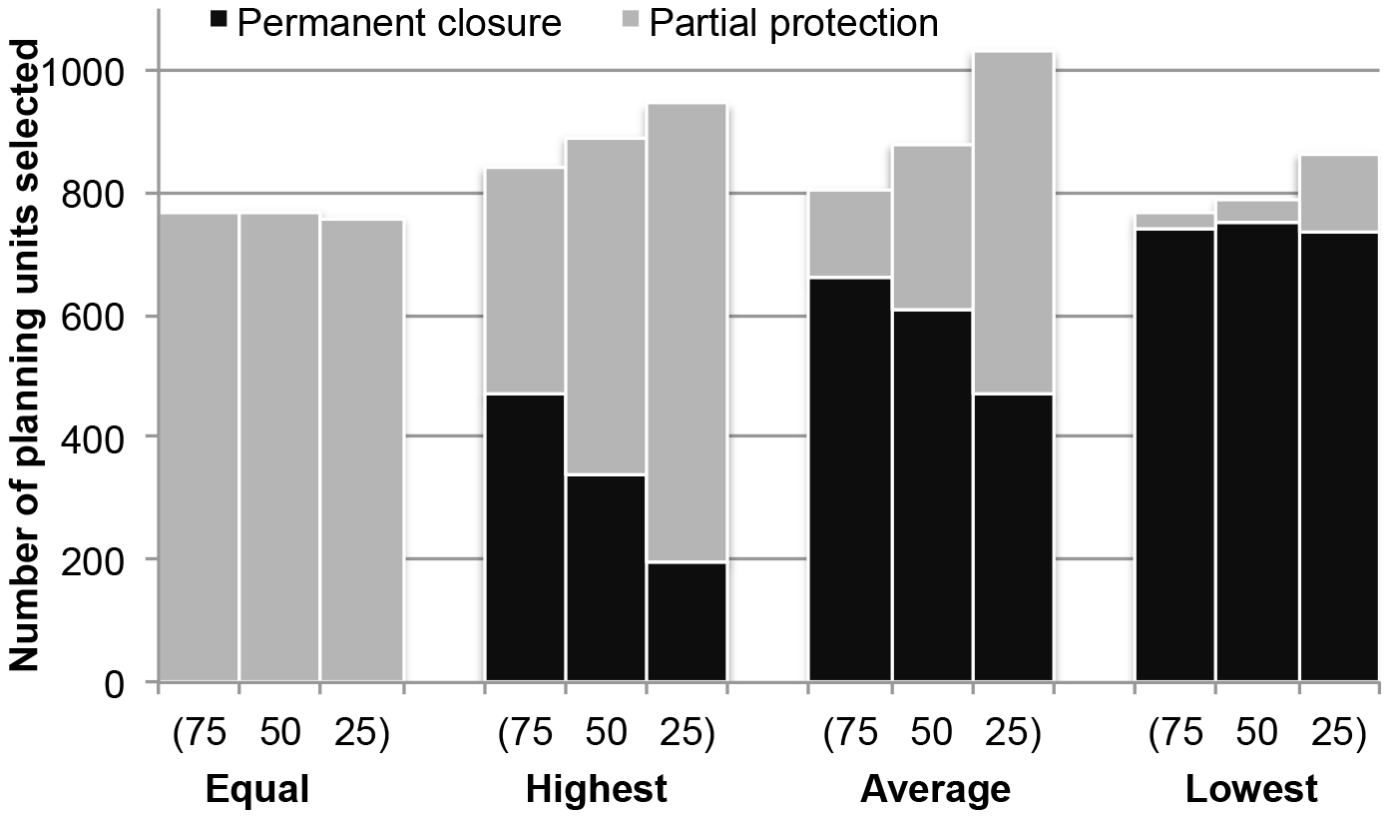
The number of planning units selected as a permanent closure and partial protection zone in the best solution. The allocation of selected planning units in the best solution (*i.e.* one solution that had the minimum score out of 100 runs) for each scenario. Scenarios used different zone effectiveness values (equal, highest, average, lowest) and relative zone costs. The numbers on the x-axis indicate the zone cost of the partial protection zone relative to the permanent closure zone (*i.e.* “75” means that the zone cost of partial protection zone is 75% of that of permanent closure zone).

The highest BIC was seen when the number of clusters was four, showing that the statistically best number of the clusters is four, which is the number of scenarios (1–4). This suggested solutions between scenarios differed statistically (Fig. 2). Regardless of zone costs, all selected planning units were allocated to the partial protection zone when the zone effectiveness of the partial protection zone equaled that of the permanent closure zone (Fig. 3). As zone effectiveness for the partial protection zone decreased, an increasing number of planning units were allocated to the permanent closure zone. Furthermore, almost all of the selected planning units were allocated to the permanent closure zone when the zone effectiveness for the partial protection zone was lowest (*i.e.* the difference between the zone effectiveness between zones was greatest).

When the cost of partial protection zones relative to permanent closure zones decreased (*i.e*. zone cost of a partial protection zone went from 75% to 25% of zone cost of a permanent closure zone), the number of planning units selected into a conservation zone (either partial protection or permanent closure zone) increased, except for when the zone effectiveness was equal for each zone (Fig. 3). The difference between zone costs influenced the proportion of planning units allocated to each zone in all cases except when the partial protection zone effectiveness was equal to the permanent closure zone effectiveness. When zone effectiveness of the partial protection zone was equal (scenario 1) or lowest (scenario 4), the relative zone cost difference did not substantially influence the number of planning units allocated to the permanent closure zone.

These trends were also seen in selection frequency of planning units. We defined planning units that were selected more than 50 times out of 100 Marxan runs as “high priority” planning units so we could compare the solutions among scenarios (Table 3). As the zone effectiveness of partial protection zones decreased, the number of high priority planning units for permanent closure zones increased and that for partial protection zones decreased for all zone costs (Table 3). When the zone effectiveness of partial protection zones at achieving conservation targets was 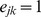 (equal to that of permanent closure zone, scenario 1), there were no high priority planning units allocated to the permanent closure zone. There were also no high priority planning units allocated to partial protection zone when the partial protection zone effectiveness was the lowest (Table 3). As the zone cost of partial protection zone varied from 75% to 25% of zone cost of permanent closure zone, the number of planning units selected into a conservation zone increased, including when the zone effectiveness was equal for each zone (Table 3).

**Table 3:** The number of planning units that were selected more than 50 times out of 100 Marxan run in scenarios (1: equal, 2: highest, 3: average, 4: lowest zone effectiveness) with the different relative zone costs of partial protection zones to permanent closure zones of 25%, 50%, and 75%.

		Scenario			
Zone	Relative zone cost	(1) Equal	(2) Highest	(3) Average	(4) Lowest
Permanent closure	25%	0	161	457	690
Partial protection	25%	711	688	414	0
Open	25%	9306	9130	9009	9183
Permanent closure	50%	0	341	594	722
Partial protection	50%	692	409	23	0
Open	50%	9318	9158	9152	9255
Permanent closure	75%	0	448	663	722
Partial protection	75%	683	91	0	0
Open	75%	9329	9221	9244	9273

This table shows the selection frequency of a planning unit out of 100 Marxan runs.

**Figure 4:**
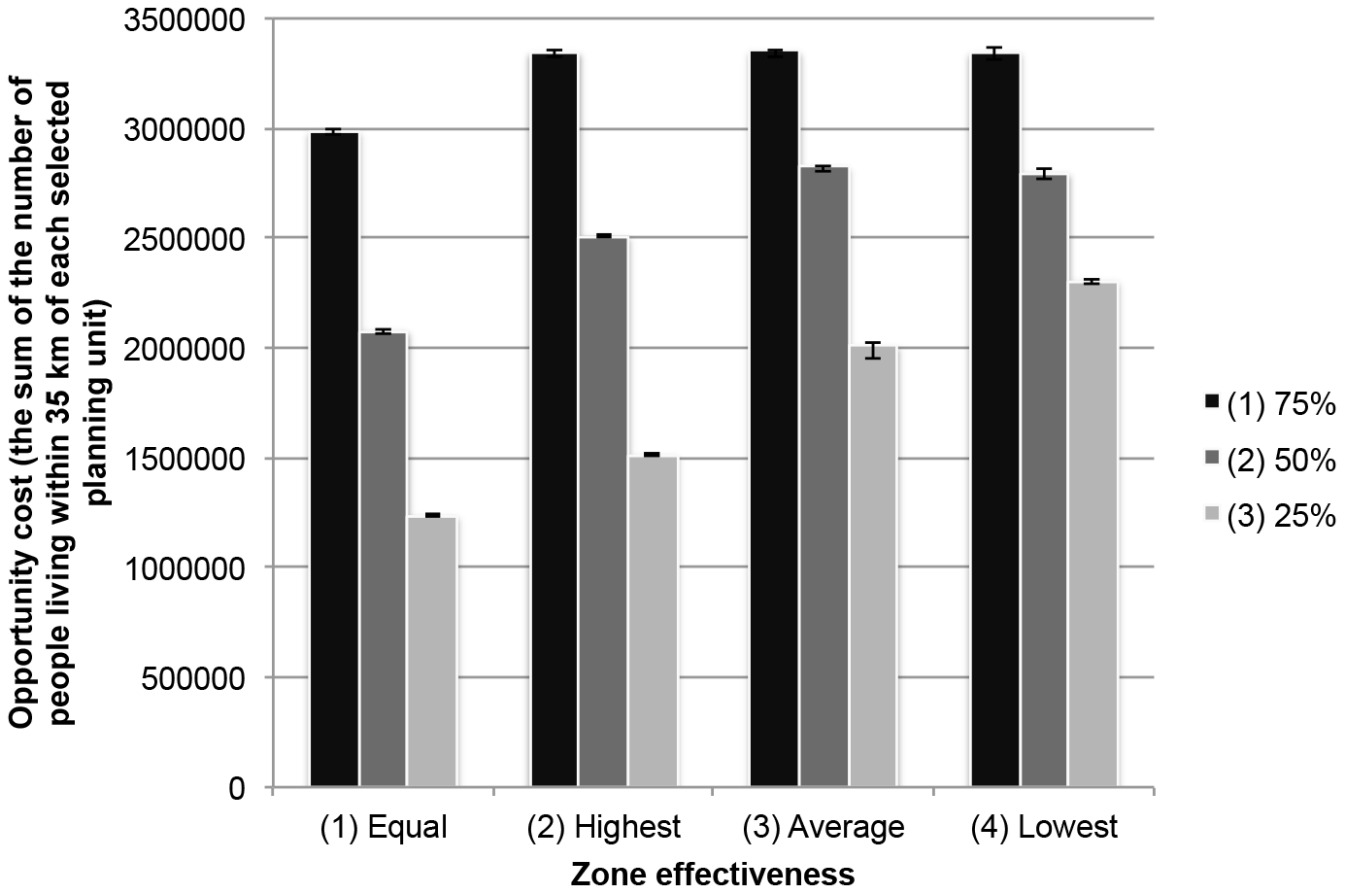
The average opportunity costs of ten best solutions of scenarios. Scenarios used different zone effectiveness values (1: equal, 2: highest, 3: average, 4: lowest zone effectiveness) with the different relative zone costs (75%, 50%, and 25%). The opportunity costs of fishing were obtained using the fishing pressure data and are the sum of the number of people living within 35 km of each planning unit.

The total opportunity cost almost doubled when zone effectiveness of the permanent closure zone and the partial protection zone were not equal (Fig. 4). The most costly solutions for marine zoning were produced when the zone effectiveness of the partial protection zone was low (scenario 4). The cost declined as the zone cost of the partial protection zone decreased relative to the permanent closure zone (Fig. 4).

When we explored the maximum equitable amount of fishing grounds remaining open for fishing while still meeting the conservation targets (scenario 5), we found 84%, 87%, 88% (for relative zone costs of partial protection zones to permanent closure zones of 25%, 50%, and 75%, respectively) of the areas of every traditional fishing ground can remain open for fishing. In contrast, in scenario 3 (average partial protection zone effectiveness), using the same zone effectiveness as in scenario 5, some traditional fishing grounds were excessively impacted because open zones for fishing were distributed inequitably. For example, without targets for fishing features (*i.e.* traditional fishing grounds), one traditional fishing ground had less than 40% of its area open for fishing while another traditional fishing ground remained entirely open for fishing. For scenario 5 where an equitable amount of fishing grounds remained open for fishing, the overall reserve system costs were 14%, 15%, and 21% (relative zone costs of partial protection zones to permanent closure zones of 25%, 50%, and 75%, respectively) greater than scenario 3 (average partial protection zone effectiveness) that used the same zone effectiveness but did not set the fishing targets.

## Discussion

Spatial prioritization traditionally assumes that a site is either inside, or outside, a protected area system. However, the ability of Marxan with Zones to place any planning unit in one of many zones opens up many options for more advanced fine-scale conservation planning [10], [12]. Here, we focused on advancing conservation zoning by exploring the impact of differential zone effectiveness and zone costs on zoning plans at a MPA network scale. We found that differential zone effectiveness and zone costs influenced the location of priorities for different zones and the allocation of planning units into a particular zone. For example, highly effective and low cost zones were more likely to be selected. As zone effectiveness and relative zone cost changed, a trade-off between zone effectiveness and cost was seen in the number of planning units allocated to permanent closure or partial protection zones. This trade-off was not seen when zone effectiveness of the partial protection zone was equal, or low, relative to the permanent closure zone because zone effectiveness became the driver for priority selection.

It is important to consider differential effectiveness between different types of conservation zones to avoid missing priority areas that need to be prudently allocated to a permanent closure zone. When we assumed that the two conservation zones were equally effective at achieving conservation targets, planning units were only selected in the most inexpensive zone, the partial protection zone. Although this could be avoided by adding a zone-specific target for the permanent closure zone (see Klein et al. [10]), it does not accurately represent the contribution of different zones to conserving biodiversity.

Differential zone effectiveness and zone costs also influenced the opportunity costs of zoning plans. As we did not have spatially explicit opportunity cost data for our analysis, we developed a surrogate to represent fishing pressure (*i.e.* opportunity cost), but acknowledge that our model may not accurately represent fishing in Fiji and that planners in other regions should seek to use actual fishing data (or other human use data) to estimate the opportunity cost of a zone. However, the consideration of opportunity costs in planning is challenging, as spatially explicit data on opportunity costs is rarely available, especially at large spatial scales although the importance of including the opportunity costs of conservation when planning for MPA is well established [27], [42]. This challenge is magnified when opportunity costs for more than one type of conservation zone are required. However, we demonstrated an approach to overcome this challenge when planning for multiple conservation zones by considering the relative cost between zones. Thus, planners can zone for multiple conservation zones with just two types of information on costs: (1) spatially explicit cost data for one zone; (2) relative difference in costs between conservation zones.

If the adverse impacts of MPAs are equitably spread amongst communities then we believe that a conservation plan is more likely to be implemented [43], [44]. However, it is important to note that the efficiency of any plan is reduced when we try to increase social and/or economic equity (*i.e.* the total cost increased when an equitable amount of fishing grounds remained open for fishing while still meeting conservation targets) [44]. This is because the inclusion of conservation zones is shared equally across communities. We do not expect communities in Fiji to exactly adopt any of our zoning plans; instead, our zoning plans can be used in conjunction with local community expertise and/or other planning processes as supporting information when developing conservation plans. For example, there currently are projects underway in Fiji to develop provincial-level zoning plans for Ra and Bua provinces, for which our technique would be appropriate when combined with initial stakeholder consultation to achieve consensus on zone type, zone effectiveness and relative costs, as well as follow-up consultations to refine placement of spatial boundaries of zones.

We demonstrated a simple approach for considering differential levels of ecological zone effectiveness when designing marine protected areas with different zones. Further, we showed how consideration of zone effectiveness affects the location and cost of spatial priorities for different zones. This approach will be informative to other places where more types of zones and human activities are being planed for, such as mining, wind farming, tourism, and other types of fisheries management zones. Such an approach would require information about the contribution of each zone to achieving conservation and industry targets, as well as the costs associated with designation of each zone. Although zone effectiveness values from experts, like those used here, are valuable, we recognize that an evaluation using empirical data would improve the robustness of values [45], [46]. In addition, quantifying the relationship between the zone effectiveness and the zone size is needed as the size of a managed area may change its overall effectiveness [47], [48]. For example, a permanent no-take area smaller than the size of the home range of targeted fish species will not provide full protection for those species [49]. Further, we did not take into account that zone effectiveness for a given zone can differ from place to place [22], [38], [39] as data were unavailable; however, given available data, this could be considered in our zoning approach. Finally, applying not only ecological effectiveness but also management effectiveness of existing closures in addition to the ecological effectiveness would improve our results.

We suggest that application of our approach should consider more realistic cost estimates for each zone. We estimated the zone cost based on a surrogate measure for foregone profit from fishing (*i.e.*, opportunity cost). Opportunity cost estimates would be improved if supported by empirical data gathered through interviews with fishermen or other fishing reports [28], [50]. Further, there are other types of financial costs associated with establishing and managing MPAs, including management and transaction costs [26], [51]. We did not consider MPA management costs because MPAs generally depend on voluntary compliance in Fiji and the relative zone costs are uncertain. Including cost information not only for fishers but also for different stakeholders would enhance our study to reflect the reality of zoning. We arbitrarily set three different relative zone costs. Assessing the zone costs using opinions from experts, empirical data or traditional ecological knowledge could improve our results [52]. Finally, our approach was static in that it did not consider the spillover effects of MPAs or the redistribution of fishing effort after protection. Incorporating these dynamic process into MPA planning is important yet complex, highlighting areas of further research.

Marine zoning requires identifying and involving stakeholders and deciding conservation objectives, the same as traditional conservation planning [53]. Recommendations and principles for designing MPAs on size, shape, connectivity and target amounts exist (*e.g.* McLeod et al. [54] and Fernandes et al. [55]). Additional guidelines are required for marine zoning. The first additional step is to list activities allowed in each zone. The activities guide table for the Great Barrier Reef Marine Park is a useful example for this stage (see http://www.gbrmpa.gov.au/zoning-permits-and-plans/zoning/zoning-maps. Accessed 30 September 2013.). The second step it to decide which zones should be included, which will depend on the conservation context. This includes clarifying the purpose of the zone, *i.e.* is it for biodiversity, fishery, customs/tradition, or industries. The third step is to assess the zone effectiveness by empirical means or expert elicitation. The fourth step is to decide how much of each feature should be represented in each type of zone. The final step is to calculate or estimate the cost of zones (*e.g.* relative cost compared with permanent zone). Although effective solutions can be produced using a systematic conservation tool, like Marxan with Zones, stakeholders should also be a part of zoning decisions [56].

Marine zoning has the ability to accommodate objectives of multiple users and minimize conflict between different resource users and stakeholders [3], [4]. However, the effects of differential effectiveness between zones had not been investigated in systematic conservation planning. In this study, we explored the impact of differential zone effectiveness on the location and costs of conservation priorities to enable more reliable conservation planning and help achieve conservation targets more effectively in Fiji. Our approach is applicable to any country, especially in places where strong traditional management practices with partial or temporary closure systems exist and permanent closure zones are unlikely to be accepted because of concerns for food security and cultural and social factors [57], [58].
